# DecOT: Bulk Deconvolution With Optimal Transport Loss Using a Single-Cell Reference

**DOI:** 10.3389/fgene.2022.825896

**Published:** 2022-02-04

**Authors:** Gan Liu, Xiuqin Liu, Liang Ma

**Affiliations:** ^1^ Department of Information and Computing Science, University of Science and Technology Beijing, Beijing, China; ^2^ Key Laboratory of Zoological Systematics and Evolution, Institute of Zoology, Chinese Academy of Sciences, Beijing, China

**Keywords:** bulk RNA sequencing, single-cell RNA sequencing, cell-type deconvolution, wasserstein distance, optimal transport

## Abstract

Tissues are constituted of heterogeneous cell types. Although single-cell RNA sequencing has paved the way to a deeper understanding of organismal cellular composition, the high cost and technical noise have prevented its wide application. As an alternative, computational deconvolution of bulk tissues can be a cost-effective solution. In this study, we propose DecOT, a deconvolution method that uses the Wasserstein distance as a loss and applies scRNA-seq data as references to characterize the cell type composition from bulk tissue RNA-seq data. The Wasserstein loss in DecOT is able to utilize additional information from gene space. DecOT also applies an ensemble framework to integrate deconvolution results from multiple individuals’ references to mitigate the individual/batch effect. By benchmarking DecOT with four recently proposed square loss-based methods on pseudo-bulk data from four different single-cell data sets and real pancreatic islet bulk samples, we show that DecOT outperforms other methods and the ensemble framework is robust to the choice of references.

## Introduction

Quantification of gene expression changes in different tissues or under different conditions gives information on how genes are regulated in organisms. The analysis of gene expression by using RNA sequencing (RNA-seq) has contributed substantially, since its development more than a decade ago, to our understanding of biological processes such as organism development, human disease progression, and patients’ response to treatments. The classic RNA-seq applied to bulk tissue samples has accumulated a rich reservoir of data sets, for example, GTEx, TCGA, and so forth (Tomczak et al., 2015, Carithers et al., 2015). However, since tissues are heterogeneous, which comprise a variety of cell types, the bulk sequencing data only measure the average state of the mixed cell populations. In fact, the information of cellular composition is crucial. For example, when developing diagnostic techniques, such information would enable researchers to track the contribution of each cellular component during disease progressions (Schelker et al., 2017).

With the rapid development of single-cell technologies, one way to obtain a cell-specific transcriptome is to apply single-cell RNA-seq (Saliba et al., 2014). However, these experiments remain costly and noisy compared to the mature bulk RNA-seq and have therefore been performed only on a limited scale (Denisenko et al., 2020): (Kuksin et al., 2021). Alternatively, one may apply computational deconvolution algorithms with bulk data, which provide cost-effective ways to derive cellular composition information and have the potential to bring considerable improvements in the speed and scale of relevant applications.

In recent years, a number of computational deconvolution methods have been developed with the goal of estimating cell-type composition within the bulk sample and/or cell-type-specific states (Avila Cobos et al., 2018); (Jin and Liu, 2021). According to whether references, such as expression profiles of pure cell types or marker gene lists, are provided, these deconvolution methods can be divided into supervised and unsupervised categories. As completely unsupervised approaches based on non-negative matrix factorization (NMF) suffer from low deconvolution accuracy and interpretation of their results largely depends on the ability to recover meaningful gene features or expression profiles for different cell types, the most commonly used methods are under the supervised category and are often optimized by least squares algorithms (Avila Cobos et al., 2018). The rapid accumulation of publicly available scRNA-seq data on a number of different samples (Baron et al., 2016), (Guo, 2020), led to the recent popularization of developing deconvolution methods with scRNA-seq references. For instance, Bisque learned the gene-specific conversion of bulk data from the scRNA-seq reference, eliminating the technical deviation of the sequencing technology between reference and bulk data (Jew et al., 2020). MuSiC proposes a weighted non-negative least squares regression framework that simultaneously weighs each gene through cross-subject and cross-cell variation (Wang et al., 2019). SCDC extends the MuSiC method and proposes an ensemble framework which applies multiple scRNA-seq data sets as reference deconvolution. They claim that SCDC can implicitly solve the batch effect between reference data sets in different experiments (Dong et al., 2019).

Besides square loss, divergence functions for characterizing differences between two distributions, for example, Kullback-Leibler divergence, are also commonly applied as loss functions in solving deconvolution problems (Lee and Seung, 1999). These losses, as well as square losses, decompose vectors or distributions in an elementwise manner, which neglects relationships between features (in our case, correlations between genes) (Zhang, 2021), (Afshar et al., 2020).

Recently, the Wasserstein distance, which originated from the optimal transport (OT) problem (Monge, 1781); (Kantorovich, 1942), has shown its potential as a better loss function for measuring the distance between distributions (Langfelder and Horvath, 2008); (Arjovsky et al., 2017). Wasserstein distance utilizes a metric between features (e.g., genes) called ground cost to take advantage of additional knowledge from the feature space (Rolet et al., 2016). Especially, when comparing two non-overlapping distributions (distributions with non-overlapping support), Wasserstein distance can still provide a smooth and meaningful measure, which is a desirable property that square loss and other divergence losses cannot offer (Weng, 2019), (Schmitz et al., 2018a). Since the first application of Wasserstein loss in solving NMF problems in Sandler and Lindenbaum, 2011, it has been successfully applied to blind source decomposition (Rolet et al., 2018), dictionary learning (Rolet et al., 2016), (Schmitz et al., 2018b), and multilabel supervised learning problems.

Cell types are characterized in gene space. The expression of genes is not mutually independent. The co-expression of genes naturally induces a similarity or distance metric among genes (Langfelder and Horvath, 2008). To the best of our knowledge, such a relationship has not yet been leveraged to solve cell-type devolution problems.

Here, we present DecOT, a bulk gene expression deconvolution method that uses the optimal transport distance as a loss and applies an ensemble framework to integrate reference information from scRNA-seq data of multiple individuals. We apply different ground cost metrics for characterizing gene relations in DecOT. We optimize DecOT under an entropic regularized form. We test the performance of DecOT on pseudo-bulk mixtures generated from different data sets and evaluate its robustness when different reference data are supplied. Finally, we applied DecOT on a real pancreatic islet bulk data set. DecOT is available on GitHub (https://github.com/lg-ustb/DecOT).

## Materials and Methods

In this section, we will first give a brief review of the original Wasserstein distance and the optimization algorithm with entropic regularization. Then, we will introduce our proposed DecOT framework for deconvolution. Finally, we will describe the data sets and procedures used for benchmarking DecOT.

### Wasserstein Distance and Entropic Regularization

Wasserstein distance, originated from the optimal transport problem (Monge, 1781); (Kantorovich, 1942), aims at minimizing transportation costs between two probability distributions. Given two histograms, $p\in {\text{Σ}}_{n}$ and $q\in {\text{Σ}}_{s}$ , the Wasserstein distance between p and q with respect to ground cost M is

$$W{\left(p,q\right)}_{M}\overset{\mathrm{def}}{=}\underset{T\in U\left(p,q\right)}{\min}<M,T>$$ (1)

where ${\text{Σ}}_{n}\overset{\mathrm{def}}{=}\left\{q\epsilon {\mathbb{R}}_{+}^{n}|<q,1>=1\right\}$ is the set of histograms or an n-dimensional simplex; $<X,Y>\overset{\mathrm{def}}{=}tr\left(X^{T}Y\right)=\sum_{i=1}^{m}X_{i},Y_{i}$ is the Frobenius dot product between matrices X and Y ; $U\left(p,q\right)=\left\{T\in {\mathbb{R}}_{+}^{n\times s}\text{|}\begin{array}{c} T1=p\\ T^{T}1=p \end{array}\right\}$ is called the transportation polytope of p and q ; M is the transportation cost of mapping p to q , which is also called the ground cost. W is a distance whenever $M_{ij}$ is a metric in these two histograms’ element space (Villani, 2009).

The computation of Wasserstein distance is extremely costly when the histograms’ dimension exceeds a few hundreds. Cuturi et al. (Cuturi, 2013) introduced an entropic regularizer to smooth the optimal transport problem, which can be computed at several orders of a magnitude faster in speed than traditional algorithms

$$W_{\gamma }{\left(p,q\right)}_{M}\overset{\mathrm{def}}{=}\underset{T\in U\left(p,q\right)}{\min}<M,T>-\gamma h\left(T\right)$$ (2)

where γ>0 is a hyperparameter. $h\left(T\right)\overset{\mathrm{def}}{=}-<T,\log T>=-\sum_{i,j}T_{ij}\text{log}\left(T_{ij}\right)$ is the entropic function.

The problem (Eq. 2) is strongly convex, and the solution of transport plan $T^{*}$ can be optimized by solving a matrix balancing problem, which is typically solved using the fixed point Sinkhorn algorithm (Sinkhorn, 1967). The hyperparameter γ plays an important role in the final performance of Sinkhorn, with higher values of γ corresponding to a faster execution of the algorithm but a more diffused coupling. In this study, unless otherwise noted, we use γ=0.001 by default.

### Cell-Type Deconvolution with Wasserstein Loss

In this section, we will introduce the bulk tissue deconvolution framework by applying the Wasserstein distance as a loss function, which is the core part of DecOT.

We assume that each cell type has a unique expression profile which can be characterized by a distribution/histogram in gene space; for instance, we denote the expression profile over n genes of cell type i as $C_{i}\in {\text{Σ}}_{n}$ . Thus, the cell type-specific profiles of k types can be represented as a k×n  matrix, $C\in {\text{Σ}}_{n}^{k}$ . For a set of normalized bulk tissue samples $\;Y=\left\{Y_{1},\ldots ,Y_{m}:Y_{j}\in {\text{Σ}}_{n},\forall j\right\}$ , the deconvolution problem is to solve the cell-type proportion or mixture proportion $P\in {\text{Σ}}_{k}^{m}$ for the m bulk samples by giving cell-type-specific profiles C , which can be represented by

Y≈C⋅P

To avoid individual/batch effects, here, we use reference data from a single individual. The annotated scRNA-seq reference data are then used by averaging the cell expressions within each cell type to generate C . The Wasserstein distance not only measures the difference between two distributions but also accounts for the underlying geometry of the feature (gene) space through the choice of an appropriate ground cost. Since the expression of genes is not mutually independent, the co-expression pattern between pairs of genes naturally induces a similarity or a distance metric among genes. Such a relationship forms the transportation cost among genes (ground cost M ) and will be incorporated in the minimization of Wasserstein distance between the bulk sample gene expression distribution Y and the estimated mixture $C\hat{P}$ . In order to ensure a trackable calculation for data containing thousands of genes, we apply the entropic regularized Wasserstein distance as a loss, which results in solving the following optimization problem

$$\underset{P\in {\text{Σ}}_{k}^{m}}{\min}{\sum_{j=1}^{m}W}_{\gamma }{\left(Y_{j},CP_{j}\right)}_{M}\;\;s.t.\;CP\in {\text{Σ}}_{n}^{m}$$ (3)

In addition, since the cell-type proportions are non-negative, we further added a regularization term, as performed by Rolet et al. (Rolet et al., 2016) in solving the dictionary learning problem with a fixed dictionary, to enforce non-negativity constraints on the variables

$$\underset{P\in {\text{Σ}}_{k}^{m}}{\min}\sum_{j=1}^{m}W_{\gamma }{\left(Y_{j},CP_{j}\right)}_{M}-\rho E\left(P_{j}\right)\;\;s.t.\;CP\in {\text{Σ}}_{n}^{m}$$ (4)

where E is defined for matrices whose columns are in the simplex as E(A)=<A,log⁡A> and ρ>0 is a hyperparameter. In this study, unless otherwise noted, we use ρ=0.001 by default.

### Ensemble Deconvolution Results Across Individuals

With the accumulation of publicly available single-cell data, references from multiple individuals may be available. In order to resolve variabilities in gene expression between references from different individuals, we adopt an ensemble approach similar to SCDC (Dong et al., 2019). The difference is that we focus on individuals rather than reference data sets of different experimental platforms. Assuming that single-cell data sets from R reference individuals are available, we first deconvolve the bulk gene expression data with entropic regularized Wasserstein loss as described above for each individual reference. Let ${\hat{C}}_{\left(r\right)}$ and ${\hat{P}}_{\left(r\right)}$ denote the cell-type-specific average expression matrix and the cell-type proportion matrix computed from the $r^{th}$ reference individual. Our goal is to find the optimal combination strategy to ensemble the available deconvolution results

$$({\hat{w}}_{1},{\hat{w}}_{2},\ldots ,{\hat{w}}_{R})=\underset{\left(w_{1},w_{2},\ldots ,w_{R}\right)}{\arg\min}l(P,\sum_{r=1}^{R}w_{r}{\hat{P}}_{\left(r\right)})$$ (5)

where l is the loss function.

As explained by Dong in SCDC (Dong et al., 2019), function (5) cannot be optimized directly since the actual cell-type proportions P are unknown, and the solutions to function (5) are approximately equivalent to minimize the loss of gene expression levels. Therefore, we change the optimization problem to

$$({\hat{w}}_{1},{\hat{w}}_{2},\ldots ,{\hat{w}}_{R})=\underset{\left(w_{1},w_{2},\ldots ,w_{R}\right)}{\text{argmin}}l(Y,\sum_{r=1}^{R}w_{r}{\hat{Y}}_{\left(r\right)})$$

where ${\hat{Y}}_{\left(r\right)}={\hat{C}}_{\left(r\right)}{\hat{P}}_{\left(r\right)}$ is the $r^{th}$ individual’s predicted bulk gene expression levels.

We redefine the problem to non-negative least squares regression by choosing the $l_{2}$ norm as loss

$$\min\|Y-\sum_{r=1}^{R}w_{r}{\hat{Y}}_{\left(r\right)}{\|}_{2}\;\;s.t.\;\sum_{r=1}^{R}w_{r}=1,\;w_{r}>0$$

Intuitively, $w_{r}$ can be seen as the similarity of cell expression profiles between $r^{th}$ reference individual and a bulk tissue-derived individual.

### Ground Cost Selection

In Wasserstein distance, a key factor is the ground cost matrix M , which defines the transportation cost. We obtain M from the reference cells an expression histogram X whose columns correspond to cells and whose rows correspond to genes. $M_{ij}$ represents the dissimilarity of expression between gene i and gene j in reference cells. Here, we focus on four metrics, including

(i) Euclidean distance: $\left|\right|x-y|{|}_{2}=\sqrt{{\sum_{i=1}^{n}\left(x_{i}-y_{i}\right)}^{2}}$ .
(ii) Cosine similarity: $\cos\left(x,y\right)=\frac{xy}{\left|\right|{\mathrm{x||}}_{2}\times {\mathrm{||y||}}_{2}}$ . We use 1−cos(x,y) as distance.
(iii) Pearson correlation: $\text{cor}\left(x,y\right)=\cos\left(x-\bar{x},y-\bar{y}\right)$ , where $\bar{x}$ and $\bar{y}$ are the mean of the values of x and y , respectively. We use 1−cor(x,y)  as distance.
(iv) Topological overlap-based dissimilarity measure (dissTOM) (Ravasz et al., 2002; Li and Horvath, 2007; Yip and Horvath, 2007) underweighted gene co-expression network analysis framework (Zhang and Horvath, 2005)

$$d_{ij}^{\omega }=1-\frac{\sum_{u}a_{iu}a_{uj}+a_{ij}}{\min\left\{\sum_{u}a_{iu},\sum_{u}a_{ju}\right\}+1-a_{ij}}$$

where $a_{ij}$ is the power adjacency function. dissTOM metric measures the distance between genes in a co-expression network, which is converted into a scale-free network. We use a python package POT (Flamary et al., 2021) to compute metrics (i)–(iii) and WGCNA (Langfelder and Horvath, 2008)..., a R package ... to compute dissTOM.

### Benchmark Data Sets and Artificial Pseudo-bulk Mixtures

To evaluate DecOT and compare it to other deconvolution methods using $l_{2}$ norm loss, we generated artificial pseudo-bulk mixtures from four real RNA-seq data sets (see Table 1). We partly adopt the preprocessing and quality control pipeline in Cobos et al. (Avila Cobos et al., 2020) to the original data, which include filtering genes with all zero expression or zero variance, removing cells with the library size deviating from the mean size over three median absolute deviations (MADs), keeping genes with at least 5% of all cells having a UMI or read count greater than 1, and retaining cell types with at least 50 cells passing the quality control step (Avila Cobos et al., 2020).

**TABLE 1:** Four real scRNA-seq data sets.

Data set	Tissue type	Data type	Protocol	Individual samples	Cells	Genes	Cell types
Baron (GSE84133) Baron et al. (2016)	Pancreatic islet	Single-cell RNA-seq	Illumina HiSeq 2,500 (InDrop)	4	7,876	8,415	10
E-MTAB-5061 Segerstolpe et al. (2016)	Pancreatic islet	Single-cell RNA-seq	Smart-seq2	10	1901	14,200	7
GSE81547 Enge et al. (2017)	Pancreatic islet	Single-cell RNA-seq	Smart-seq2	8	2073	11,861	5
Kidney.HCL Han et al. (2020) Guo, (2020)	Kidney	Single-cell RNA-seq	Microwell-seq	3	20,601	2,748	13

After quality control, for each individual in each data set, we split their cells evenly into the reference set and testing set with similar distribution of cell types. Then, we generate 200 pseudo-bulk mixtures by randomly sampling 60% of the cells each time in testing data sets and aggregate the expression counts of each gene to generate the pseudo-bulk sample. The true cell-type proportions are recorded, which allows us to benchmark the performance of different deconvolution methods. The flow chart for constructing pseudo-bulk mixtures is shown in Supplementary Figure S1.

To evaluate the performance of deconvolution methods, we need to measure the deviation of the estimated proportion $\hat{P}\;$ to the true P . Here, we apply the Pearson correlation coefficient and root-mean-squared error (RMSE) to evaluate the performance of deconvolution methods:

(i) Pearson correlation: $cor\left(P,\hat{P}\right)$ ;
(ii) Root-mean-squared error: RMSE = $\sqrt{\frac{1}{k\cdot m}\sum_{i}^{k}\sum_{j}^{m}{\left(P_{i,j}-{\hat{P}}_{i,j}\right)}^{2}}$ .

## Results

### Method Overview

**FIGURE 1:**
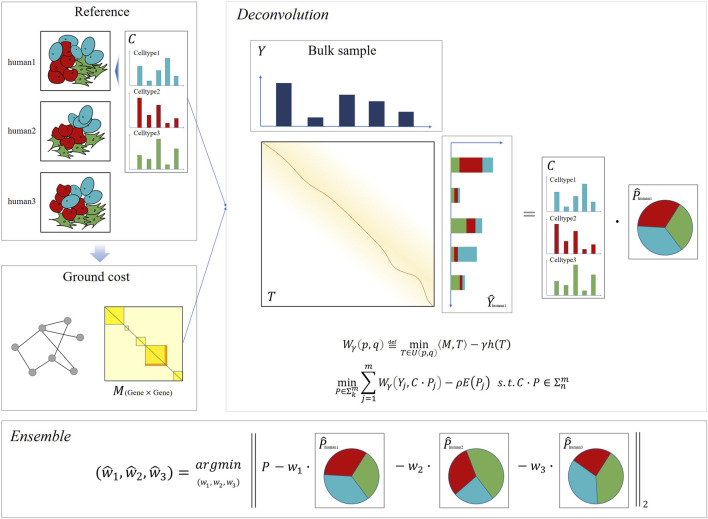
Overview of DecOT. Based on the Wasserstein loss, DecOT first converts the gene expression of single-cell and bulk samples into distributions. Next, DecOT calculates the ground cost matrix from the single-cell expression data, which forms the correlations among genes, see *Ground Cost Selection* for details. Then, for each individual reference, DecOT finds the optimal proportion of cell types by minimizing the Wasserstein loss under the premise of a given ground cost. Finally, in order to resolve the impact of batch effects between individuals, DecOT uses an ensemble framework to weigh each individual’s deconvolution result.

Since Wasserstein distance has been successfully applied to blind source decomposition (Rolet et al., 2018) and dictionary learning (Rolet et al., 2016), (Schmitz et al., 2018b) problems with excellent performance, we aimed to apply Wasserstein loss on the bulk deconvolution problem. We propose DecOT, which applies Wasserstein loss to estimate the relative abundance of cell types within a bulk sample by using a scRNA-seq reference ensemble of multi-individuals. An overview of DecOT is shown in Figure 1. DecOT first solves the entropic regularized Wasserstein loss for the cell-type deconvolution problem (*Cell Type Deconvolution with Wasserstein Loss* formula 4) based on a single individual reference constitute of scRNA-seq data with annotated cell types. Wasserstein distance aims to find the optimal transport plan under a given transportation cost. In our case, the transportation cost, also referred to as the “ground cost,” represents the similarity or distance among genes. Therefore, the application of Wasserstein loss can take advantage of the relationship between genes to get an accurate estimate.

When references from multi-individuals are available, to minimize the possible bias induced by individual and/or platform variations across different individual references, we apply an ensemble framework similar to SCDC (Dong et al., 2019), which aims to solve batch effects between reference data sets. Instead of weighting deconvolution results across a data set, DecOT seeks to optimize weights on results based on each individual reference. In this way, the individual or batch effects can be accounted for simultaneously by DecOT.

### DecOT Outperforms Deconvolution Methods Based on Squared Loss

We evaluate DecOT with different ground costs as listed in *Ground Cost Selection*, which we refer to as DecOT_dissTOM, DecOT_euclidean, DecOT_cosine, and DecOT_correlation. For these four settings, we apply the aggregated reference, which is, pooling cells from multiple individuals to generate a single reference. In addition, we also evaluate DecOT with dissTOM under the ensemble framework (referred to as DecOT_disTOM_ensamble). The various settings of DecOT are then compared to four other square loss-based methods (including Nonnegative least squares (NNLS), MuSiC, SCDC, and Bisque) on artificial pseudo-bulk mixtures generated from four scRNA-seq data sets (Table 1, Methods). Since it is possible by design to assay both bulk-RNA and scRNA from the same individual (Kuksin et al., 2021), we consider settings of reference data in two situations:

a) There are annotated single-cell reference data from the same individual, from which the bulk sample is collected. We term such a situation as “paired”.
b) Reference data are all collected from other individuals. We refer to such a scenario as “unpaired”.

We mimic the “paired” situations in the benchmark by including cells (in the reference set) from the same individual for generating a pseudo-bulk sample (in the testing set) (Supplementary Figure S1).

**FIGURE 2:**
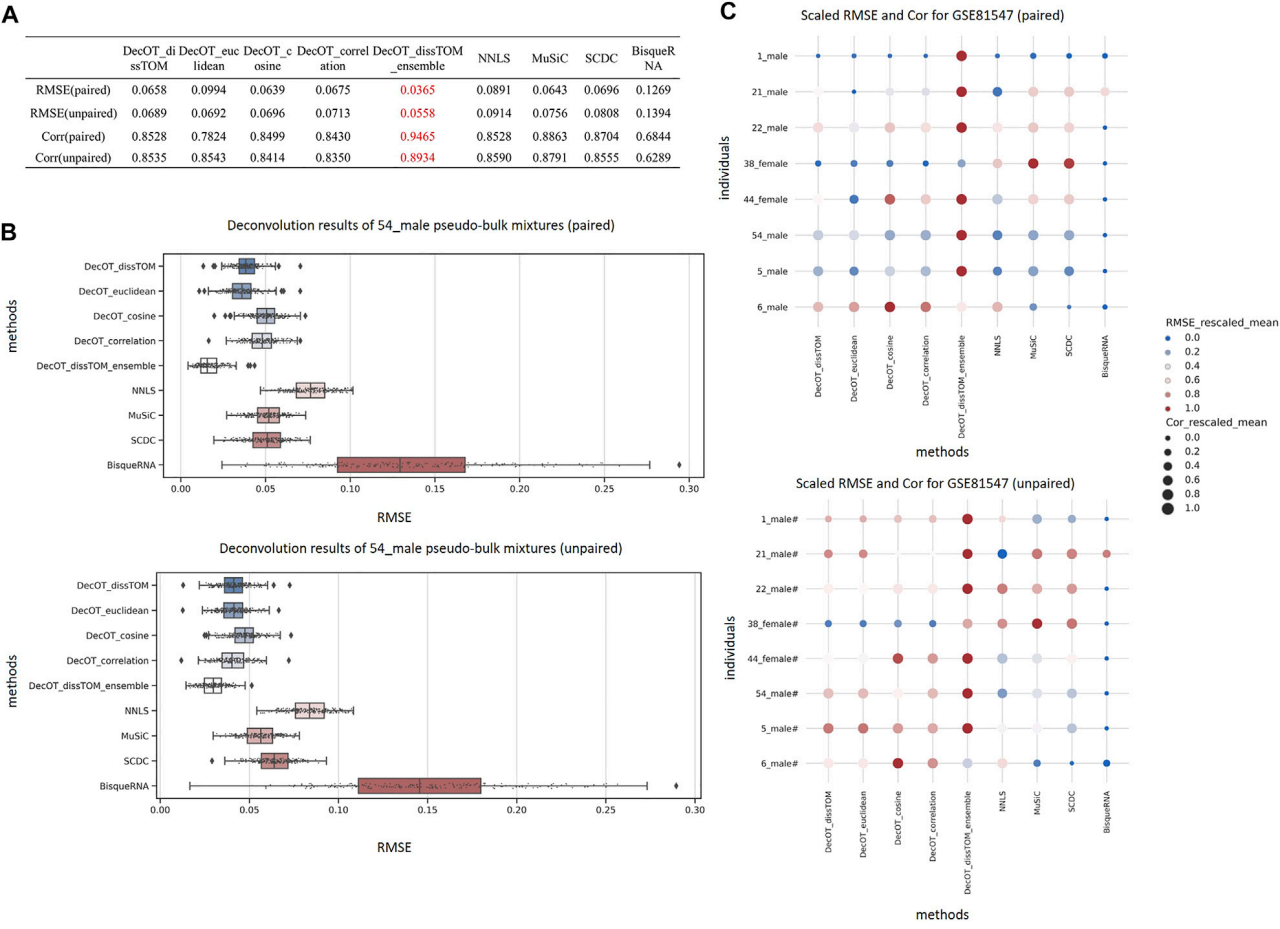
Benchmark results using data set GSE81547. **(A)** Average RMSE and Cor of the deconvolution results of all mixtures in data set GSE81547. From the overall results, the estimation of DecOT with the ensemble framework has smaller errors and stronger correlation than other methods. **(B)** Boxplot of RMSE from 200 replicate pseudo-bulk mixtures from sample 54_male. The top/bottom panel shows the results under paired/unpaired situation. **(C)** Overview of deconvolution results of individual pseudo-bulk mixtures across all methods in data set GSE81547. For each individual, we rank the results across different methods and rescale them to the interval between 0 and 1. A darker-red and larger point within a line represents a smaller RMSE and a larger Cor. Both paired (top) and unpaired (bottom) situations are considered.

Figure 2 shows the benchmark result of data set GSE81547 from Enge et al. (Enge et al., 2017) under these two situations. Applying DecOT under the ensemble framework has the best overall performance compared to other settings and methods. The average RMSE of DecOT_dissTOM_ensemble over all pseudo-bulks is 0.037 and 0.056 under paired and unpaired situations, respectively, and the average correlation is 0.946 and 0.893 (Figure 2A). Figure 2B shows the detailed estimation results of individual sample 54_male in GSE81547. DecOT with an ensemble framework using dissTOM shows the greatest performance. Even when applying aggregated references, Wasserstein’s loss still outperforms NNLS.

In order to show the overall quality of the various methods in pseudo-bulk mixtures generated from different samples in GSE81547, we compared the mean RMSEs and mean Cors, which result from performing different methods on the pseudo-bulk generated based on different individuals (Figure 2C). For each individual, we rank the results across different methods and rescale them to the interval between 0 and 1. As shown in Figure 2C, the dark-red and larger points within a line represent a smaller RMSE and a larger Cor. In general, DecOT using Wasserstein loss has better performance than square loss methods in most cases, and the ensemble framework can further improve the accuracy of the deconvolution results even when the mixtures and reference cells come from different individuals.

Similar conclusions are also obtained from benchmarks based on the other three data sets. The results are shown in Supplementary Figures S2–S4.

### DecOT Performs Robustly Under the Ensemble Framework

**FIGURE 3:**
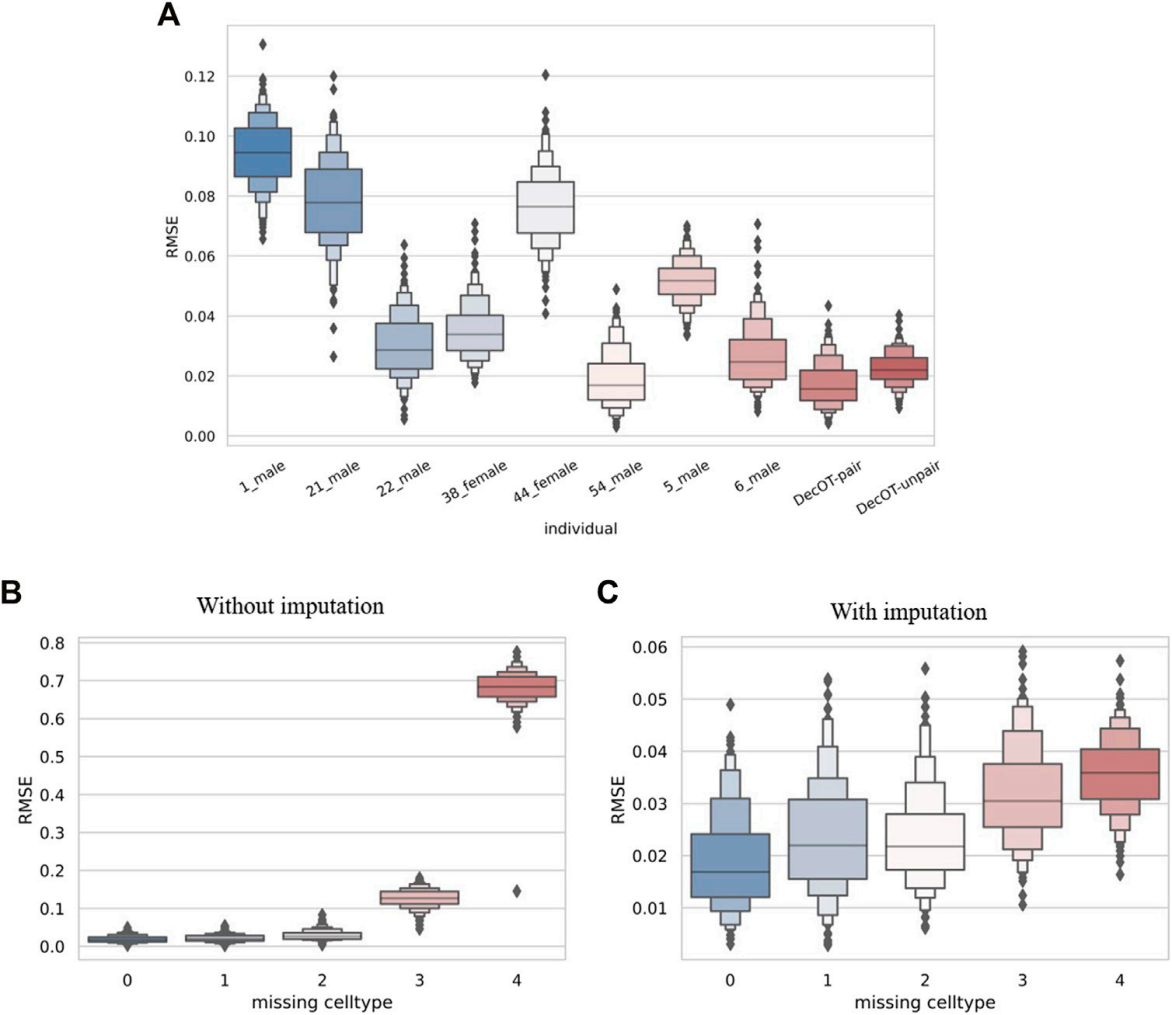
Effects under different manipulation references benchmarked by mixtures constructed from 54_male of GSE81547. **(A)** Comparison of the results from single individual references and multi-individual references under the ensemble framework. **(B,C)** Deconvolution results with missing cell types in paired references. The cell types are progressively removed according to the ascending order of cell counts in 54_male. **(B)** Direct application of the paired reference from 54_male with the missing cell type. **(C)** Application of the paired reference from 54_male with missing cell types imputed by references from other individuals.

The choice of reference in solving the supervised deconvolution problem is crucial. We first compare the performance of DecOT by using references from different individuals. In detail, we evaluate DecOT on the pseudo-bulk generated from the testing set of 54_male in GSE81547 by respectively applying reference data from each individual as well as under the ensemble framework (paired and unpaired). Figure 3A shows the result out of 200 pseudo-bulk mixtures in each reference setting. Using references from the same individual (reference set from 54_male) outperforms the situation of applying references from other individuals (Figure 3A). The deconvolution performance is slightly improved with integrating results across all individuals (paired), indicating that the DecOT ensemble framework makes use of information from other individuals to adjust the final estimation. Such a finding is further confirmed in the case under the unpaired reference situation; when excluding 54_male from the reference, the estimation of DecOT under the ensemble framework still obtains a smaller error than using other single individual references. In fact, including more individual references under the ensemble framework tends to improve the performance of deconvolution (Supplementary Figure S5).

Deconvolution with paired single-cell data as a reference will greatly improve the performance. However, in a more realistic scenario, single cells collected from the same individual may have missing cell types as compared to the paired bulk sample, especially when the cell type is rare. Therefore, we conducted an experiment by gradually and cumulatively removing cell types in ascending order of cell count in the reference set of 54_male (Supplementary Table S1) and used the data with the missing cell type as a reference. When there is a missing cell type in the reference, the deconvolution may allocate the expression of the missing cell type to other types, which leads to biased estimation (Figure 3B). One way to reduce such bias is to impute the missing cell type in the reference by utilizing a publicly available data set as a surrogate. Here, we use the mean expression of the missing cell type from references of other individuals for imputation (Figure 3C). Compared to the results in Figures 3B,C, imputation of missing cell types significantly improves the performance of deconvolution. Nevertheless, regardless of imputation, the estimation error will get worse as the number of missing cell types increases.

Another possible way for reducing the impact caused by missing cell types in paired single-cell references is to apply DecOT under the ensemble framework. Since our ensemble framework integrates deconvolution results respectively performed under each individual reference, we can still apply imputation on missing cell types in the paired reference. Table 2 compares the average RMSE of cases based on single references from paired single-cell data (RMSE-54_male) and ensemble references which account all possible individuals (RMSE-ensemble). In addition, we use the unpaired ensemble case as a baseline. The weight contributions of references from each individual are also displayed in Table 2. Since the pseudo-bulk mixtures are constructed from 54_male, the reference from one’s own cell (self-ref) contributed the most to the ensemble result. The weight contribution from self-ref decreases with the increasing number of missing cell types. The ensemble DecOT estimation under the ensemble framework is always better than using a single reference, even though it is collected from the same individual as for the bulk sample. Such a result verifies that the ensemble framework can integrate the information of multiple individuals to get a better estimate even if there is a cell type missing in the paired reference. In general, the results from the ensemble framework are rather robust under missing cell types in paired references (regardless of whether they are imputed or not).

**TABLE 2:** Optimal weights of different individual references under the DecOT ensemble framework. The weights and the overall performance are compared under different settings of the missing cell type in the paired reference of sample 54_male. Imputation indicates that the reference profiles of missing types are imputed by references from other individuals.

	Optimal weight with imputation								RMSE-54_male	RMSE-ensemble
	1_male	21_male	22_male	38_female	44_female	54_male	5_male	6_male		
54_male-all	0.0000	0.0000	0.1654	0.0000	0.0000	0.7504	0.0842	0.0000	0.0190	0.0175
54_male-delta	0.0000	0.0000	0.1691	0.0000	0.0000	0.7412	0.0817	0.0081	0.0234	0.0215
54_male-delta-ductal	0.0000	0.0000	0.1772	0.0000	0.0000	0.7293	0.0830	0.0104	0.0234	0.0218
54_male-delta-ductal-acinar	0.0000	0.0000	0.1684	0.0000	0.0000	0.6985	0.0934	0.0397	0.0318	0.0289
54_male-delta-ductal-acinar-beta	0.0000	0.0000	0.1765	0.0000	0.0475	0.6251	0.1509	0.0000	0.0359	0.0306
54_male-unpair	0.0000	0.0000	0.5114	0.0000	0.1837	—	0.1487	0.1561	—	0.0227

	**Optimal weight without imputation**								**RMSE-54_male**	**RMSE-ensemble**
	**1_male**	**21_male**	**22_male**	**38_female**	**44_female**	**54_male**	**5_male**	**6_male**		
54_male-all	0.0000	0.0000	0.1462	0.0000	0.0000	0.7483	0.0859	0.0196	0.0190	0.0172
54_male-delta	0.0000	0.0000	0.1558	0.0000	0.0000	0.7310	0.0901	0.0231	0.0211	0.0165
54_male-delta-ductal	0.0000	0.0000	0.1625	0.0000	0.0000	0.7069	0.1093	0.0212	0.0293	0.0237
54_male-delta-ductal-acinar	0.0000	0.0000	0.2971	0.0000	0.0545	0.3624	0.2173	0.0686	0.1268	0.0444
54_male-delta-ductal-acinar-beta	0.0000	0.0000	0.4654	0.0000	0.1840	0.0081	0.1670	0.1755	0.6834	0.0226
54_male-unpair	0.0000	0.0000	0.4692	0.0000	0.1855	—	0.1683	0.1770	—	0.0224

### Performance of DecOT on Human Pancreatic Islet Data

**FIGURE 4:**
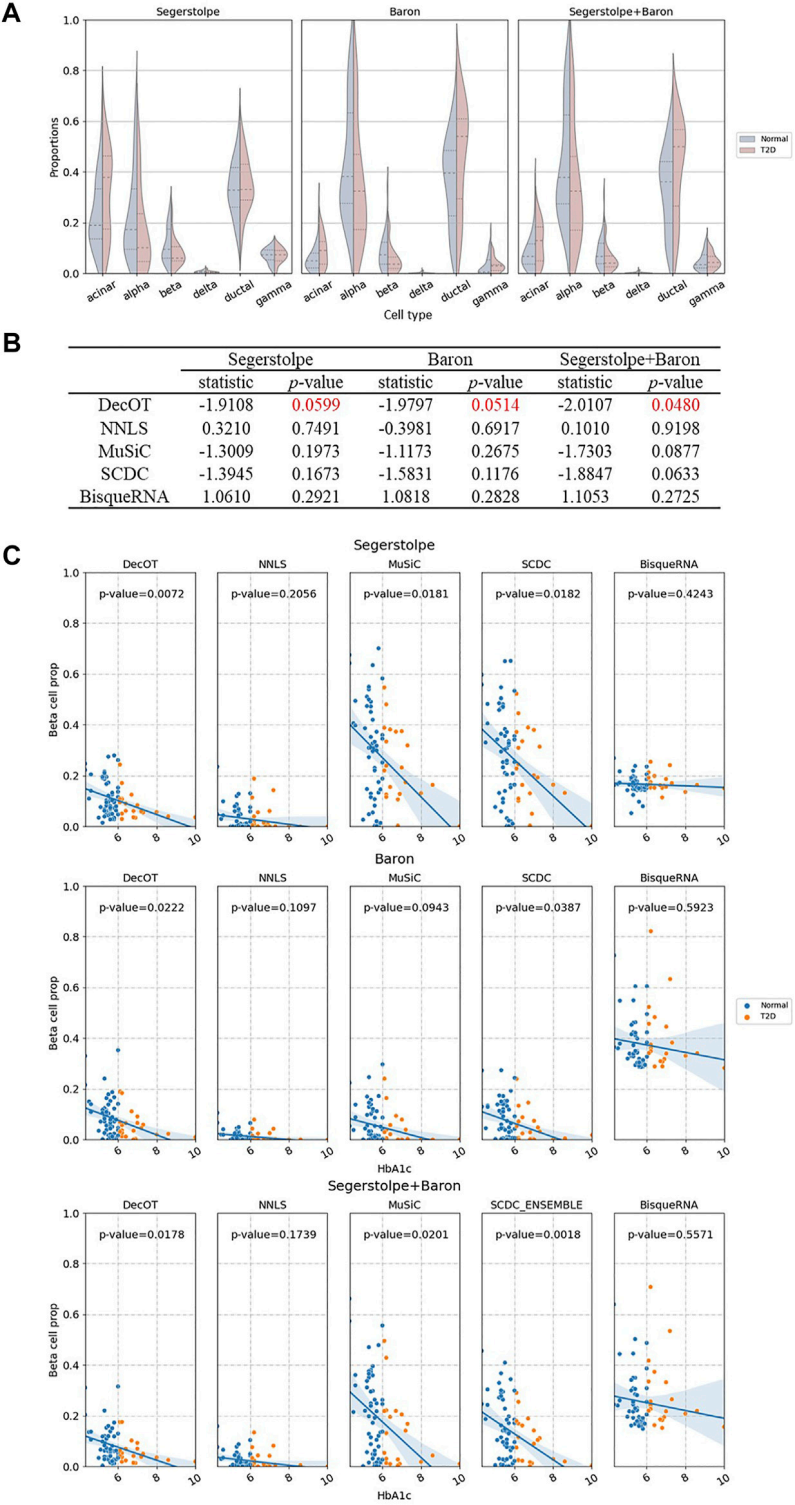
Cell-type deconvolution of healthy and T2D human pancreatic islet samples. **(A)** Estimated composition of islet cell types in healthy and type 2 diabetes (T2D) humans by DecOT under three settings of references. The violin plots show the proportion differences between healthy and T2D samples. **(B)** Independent sample t-tests of beta cell proportion between healthy and T2D individuals. DecOT shows the most significant difference as compared to other methods. **(C)** Linear regression of HbA1c expression level and the proportion of beta cells estimated by five methods. The reported *p*-values come from a multivariate linear regression model: beta cell ratio ∼ HbA1c + age + BMI + gender.

Next, we apply DecOT with dissTOM as the ground cost to deconvolve the bulk samples of 89 human islets from Fadista et al. (Fadista et al., 2014), which contains 51 healthy individuals, 26 type 2 diabetic (T2D) individuals, and 12 unknown individuals. We focus on the composition of six cell types of interest (alpha, beta, delta, gamma, acinar, and ductal) in the human pancreatic islet. We use three groups of scRNA-seq references, denoted as the Baron reference (Avila Cobos et al., 2020), Segerstolpe reference (Segerstolpe et al., 2016), and ensemble reference, which combine data from both studies. Figure 4A shows the deconvolution results of DecOT on the six types of cells by contrasting the status of individuals (normal or T2D). The proportion of beta cells that secrete insulin will gradually decrease with the progression of type 2 diabetes (T2D) (Kanat et al., 2011), (Hou et al., 2015). DecOT can successfully detect such a proportion difference between normal and T2D patients, regardless of which group of reference is used for analysis. In addition, we also apply independent sample t-tests on the beta cell proportion estimated by DecOT between normal and T2D groups. The estimates of DecOT based on all three reference groups all result in significant differences in beta cell proportion between normal and T2D samples (Figure 4B). When comparing the results with those of the four other deconvolution methods, DecOT shows the most significant *p*-values (Figure 4B). Note that for the ensemble reference, SCDC applies its built-in ENSEMBLE method, which weighs the deconvolution results across two sources of references. The other methods directly use the pooled data as references.

Previous studies have shown that in human pancreatic islet samples, hemoglobin A1c (HbA1c) is an important biomarker of type 2 diabetes, and its expression level should be negatively correlated with beta cell functions (Kanat et al., 2011), (Hou et al., 2015), (Frogner et al., 2015). We perform linear regression to the estimates of beta cell proportion (BP) by HbA1c and adding age, gender, and BMI as covariates. Figure 4C shows the regression results. The estimates of BP by NNLS and BisqueRNA failed to recover a significant negative correlation to the level of HbA1c. The beta cell proportion estimated by DecOT, MuSiC, and SCDC based on the three groups of references discovered significant negative correlations with HbA1c. When using a single-source reference, DecOT calculated the smallest *p*-values (0.0599 and 0.0514), indicating a more significant correlation between BP and HbA1c levels. In fact, the estimated BP by DecOT is robust over all three groups of references, which can be seen from the variation between the slops of the fitted regression line in Figure 4C. In contrast, the slopes have greater differences in MuSiC and SCDC cases when a different reference is applied. In short, DecOT shows better performance on real data sets and is robust to different sources of references.

## Discussion

In this study, we proposed DecOT, which applies single-cell data as references and uses Wasserstein distance as a loss function for decomposing bulk cell types. Compared with the commonly used square loss methods, the optimization of Wasserstein loss in DecOT is able to utilize additional information from gene space, for example, ground cost induced by gene-gene relations. By benchmarking DecOT with four recently proposed square loss-based methods on pseudo-bulk data from four different single-cell data sets and real pancreatic islet bulk samples, DecOT shows superior performance.

Wasserstein loss accounts for the distance between genes through the ground cost matrix. In this study, we evaluated four possible choices of ground cost, namely, three common metrics (Euclidean distance, cosine similarity, and Pearson correlation) and the dissTOM distance based on gene co-expression networks. In the analysis of simulated data, the final deconvolution effect of the four metrics did not show much difference; however, since the topological overlap measure (TOM) has been considered a more robust measure of gene interconnections (Li and Horvath, 2007), we recommend using dissTOM over other metrics.

Although DecOT obtains better deconvolution accuracy by using Wasserstein loss, optimization of such a loss also brings a greater computational cost. The application of entropic regularization allows tractable computation of data sets on a larger scale. However, there is a trade-off between accuracy and computation time. This trade-off can be tuned by the two hyperparameters γ and ρ . In Supplementary Figure S6, we show the calculation time of DecOT under different numbers of genes and the accuracy and time of DecOT calculations under different choices of two regularization parameters. We show that the performance of DecOT is rather robust with parameters in the range of γ≤0.05 and ρ≤0.01 , which results in higher calculation accuracy.

When applying a supervised bulk-cell-type deconvolution algorithm, the possible individual variation and batch effect should be noted when combining references from multiple individuals and/or data sets. DecOT uses an ensemble framework to weigh the deconvolution across multiple results from each individual reference to mitigate individual effects. The weights of the ensemble framework indicate, to a certain extent, the similarity of the gene distribution between the reference individuals and the bulk samples. In the benchmarks on pseudo-bulk data, DecOT using the ensemble framework shows improved accuracy and robustness over existing methods in most scenarios.

The performance of deconvolution will be greatly improved when paired single-cell references are available. However, there can be a problem regarding the cell-type integrity in the paired reference. We have tested two solutions in the study, imputation of the missing cell types, and/or applying the ensemble framework with DecOT. The results show that the ensemble framework can effectively utilize information of missing cell types from other reference individuals by adjusting the weights. Although the imputation solution also achieves acceptable results, the ensemble framework of DecOT shows more robust performance.

## Data Availability

All the data sets used for this study can be found at GitHub: https://github.com/lg-ustb/DecOT. These data sets are downloaded from their respective sources: GSE84133, GSE81547, E-MTAB-5061, GSE134355, and GSE50398 and https://figshare.com/articles/HCL_DGE_Data/7235471.

## References

[B1] Afshar A. Yin K. Yan S. Qian C. Ho J. C. Park H. (2020). Swift: Scalable Wasserstein Factorization for Sparse Nonnegative Tensors. arXiv preprint arXiv:2010.04081.

[B2] Arjovsky M. Chintala S. Bottou L. (2017). “Wasserstein Generative Adversarial Networks,” in International Conference on Machine Learning (PMLR), Sydney, Australia, 214–223.

[B3] Avila Cobos F. Alquicira-Hernandez J. Powell J. E. Mestdagh P. De Preter K. (2020). Benchmarking of Cell Type Deconvolution Pipelines for Transcriptomics Data. Nat. Commun. 11 (1), 1–14. 10.1038/s41467-020-19015-1 33159064 PMC7648640

[B4] Avila Cobos F. Vandesompele J. Mestdagh P. De Preter K. (2018). Computational Deconvolution of Transcriptomics Data from Mixed Cell Populations. Bioinformatics 34 (11), 1969–1979. 10.1093/bioinformatics/bty019 29351586

[B5] Baron M. Veres A. Wolock S. L. Faust A. L. Gaujoux R. Vetere A. (2016). A Single-Cell Transcriptomic Map of the Human and Mouse Pancreas Reveals Inter- and Intra-cell Population Structure. Cel Syst. 3 (4), 346–360. 10.1016/j.cels.2016.08.011 PMC5228327 27667365

[B6] Carithers L. J. Moore H. M. Salvatore M. Phillips R. Lo E. Shad S. (2015). The Genotype-Tissue Expression (GTEx) Project. Biopreservation and Biobanking 13 (6), 307–308. 10.1089/bio.2015.29031.hmm 26484569 PMC4692118

[B7] Cuturi M. (2013). Sinkhorn Distances: Lightspeed Computation of Optimal Transport. Adv. Neural Inf. Process. Syst. 26, 2292–2300.

[B8] Denisenko E. Guo B. B. Jones M. Hou R. de Kock L. Lassmann T. (2020). Systematic Assessment of Tissue Dissociation and Storage Biases in Single-Cell and Single-Nucleus RNA-Seq Workflows. Genome Biol. 21 (1), 130. 10.1186/s13059-020-02048-6 32487174 PMC7265231

[B9] Dong M. Thennavan A. Urrutia E. Li Y. Perou C. M. Zou F. (2019). SCDC: Bulk Gene Expression Deconvolution by Multiple Single-Cell RNA Sequencing References. 10.1101/743591 PMC7820884 31925417

[B10] Enge M. Arda H. E. Mignardi M. Beausang J. Bottino R. Kim S. K. (2017). Single-cell Analysis of Human Pancreas Reveals Transcriptional Signatures of Aging and Somatic Mutation Patterns. Cell 171, 321–330. 10.1016/j.cell.2017.09.004 28965763 PMC6047899

[B11] Fadista J. Vikman P. Laakso E. O. Mollet I. G. Esguerra J. L. Taneera J. (2014). Global Genomic and Transcriptomic Analysis of Human Pancreatic Islets Reveals Novel Genes Influencing Glucose Metabolism. Proc. Natl. Acad. Sci. 111 (38), 13924–13929. 10.1073/pnas.1402665111 25201977 PMC4183326

[B12] Flamary R. Courty N. Gramfort A. Alaya M. Z. Boisbunon A. Chambon S. (2021). Pot: Python Optimal Transport. J. Machine Learn. Res. 22 (78), 1–8.

[B13] Frogner C. Zhang C. Mobahi H. Araya-Polo M. Poggio T. (2015). Learning with a Wasserstein Loss. arXiv preprint arXiv:1506.05439.

[B14] Guo G. (2020). HCL DGE Data.

[B15] Han X. Zhou Z. Fei L. Sun H. Wang R. Chen Y. (2020). Construction of a Human Cell Landscape at Single-Cell Level. Nature 581 (7808), 303–309. 10.1038/s41586-020-2157-4 32214235

[B16] Hou X. Liu J. Song J. Wang C. Liang K. Sun Y. (2015). Relationship of Hemoglobin A1c withβCell Function and Insulin Resistance in Newly Diagnosed and Drug Naive Type 2 Diabetes Patients. J. Diabetes Res. 2016 (2015-11-10), 1–6. 10.1155/2016/8797316 PMC4657079 26640807

[B17] Jew B. Alvarez M. Rahmani E. Miao Z. Ko A. Garske K. M. (2020). Accurate Estimation of Cell Composition in Bulk Expression through Robust Integration of Single-Cell Information. Nat. Commun. 11 (1). 10.1038/s41467-020-15816-6 PMC7181686 32332754

[B18] Jin H. Liu Z. (2021). A Benchmark for RNA-Seq Deconvolution Analysis under Dynamic Testing Environments. Genome Biol. 22 (1), 1–23. 10.1186/s13059-021-02290-6 33845875 PMC8042713

[B19] Kanat M. Winnier D. Norton L. Arar N. Jenkinson C. Defronzo R. A. (2011). The Relationship between β-Cell Function and Glycated Hemoglobin. Diabetes Care 34 (4), 1006–1010. 10.2337/dc10-1352 21346184 PMC3064013

[B20] Kantorovich L. V. (1942). On the Transfer of Masses. review of politics.

[B21] Kuksin M. Morel D. Aglave M. Danlos F.-X. Marabelle A. Zinovyev A. (2021). Applications of Single-Cell and Bulk Rna Sequencing in Onco-Immunology. Eur. J. Cancer 149, 193–210. 10.1016/j.ejca.2021.03.005 33866228

[B22] Langfelder P. Horvath S. (2008). Wgcna: an R Package for Weighted Correlation Network Analysis. Bmc Bioinformatics 9 (1), 559. 10.1186/1471-2105-9-559 19114008 PMC2631488

[B23] Lee D. D. Seung H. S. (1999). Learning the Parts of Objects by Non-negative Matrix Factorization. Nature 401 (6755), 788–791. 10.1038/44565 10548103

[B24] Li A. Horvath S. (2007). Network Neighborhood Analysis with the Multi-Node Topological Overlap Measure. Bioinformatics 23, 222–231. 10.1093/bioinformatics/btl581 17110366

[B25] Monge G. (1781). Mémoire sur la théorie des déblais et des remblais.

[B26] Ravasz E. Somera A. L. Mongru D. A. Oltvai Z. N. Barabási A-L. (2002). Hierarchical Organization of Modularity in Metabolic Networks. Science 297 (5586), 1551–1555. 10.1126/science.1073374 12202830

[B27] Rolet A. Cuturi M. Peyré G. (2016). “Fast Dictionary Learning with a Smoothed Wasserstein Loss” in Artificial Intelligence and Statistics (PMLR), Cadiz, Spain, 630–638.

[B28] Rolet A. Seguy V. Blondel M. Sawada H. (2018). Blind Source Separation with Optimal Transport Non-negative Matrix Factorization. EURASIP J. Adv. Signal. Process. 2018. 10.1186/s13634-018-0576-2

[B29] Saliba A.-E. Westermann A. J. Gorski S. A. Vogel J. (2014). Single-cell RNA-Seq: Advances and Future Challenges. Nucleic Acids Res. 42 (14), 8845–8860. 10.1186/1755-8794-4-5410.1093/nar/gku555 25053837 PMC4132710

[B30] Sandler R. Lindenbaum M. (2011). Nonnegative Matrix Factorization with Earth Mover's Distance Metric for Image Analysis. IEEE Trans. Pattern Anal. Mach. Intell. 33 (8), 1590–1602. 10.1109/tpami.2011.18 21263163

[B31] Schelker M. Feau S. Du J. Ranu N. Klipp E. MacBeath G. Schoeberl B. Raue A. (2017). Estimation of Immune Cell Content in Tumour Tissue Using Single-Cell RNA-Seq Data. Nat. Commun. 8 (1), 1–12. 10.1038/s41467-017-02289-3 29230012 PMC5725570

[B32] Schmitz M. A. Heitz M. Bonneel N. Ngolè F. Coeurjolly D. Cuturi M. (2018). Wasserstein Dictionary Learning: Optimal Transport-Based Unsupervised Nonlinear Dictionary Learning. SIAM J. Imaging Sci. 11 (1), 643–678. 10.1137/17m1140431

[B33] Schmitz M. A. Heitz M. Bonneel N. Ngolè F. Coeurjolly D. Cuturi M. (2018). Wasserstein Dictionary Learning: Optimal Transport-Based Unsupervised Nonlinear Dictionary Learning. SIAM J. Imaging Sci. 11 (1), 643–678. 10.1137/17M1140431

[B34] Segerstolpe Å Palasantza A. Eliasson P. Andersson E.-M. Andréasson A.-C. Sun X. (2016). Single-cell Transcriptome Profiling of Human Pancreatic Islets in Health and Type 2 Diabetes. Cel Metab. 24, 593–607. 10.1016/j.cmet.2016.08.020 PMC5069352 27667667

[B35] Sinkhorn R. (1967). Diagonal Equivalence to Matrices with Prescribed Row and Column Sums. The Am. Math. Monthly 74 (4), 402–405. 10.2307/2314570

[B36] Tomczak K. Czerwińska P. Wiznerowicz M. (2015). Review the Cancer Genome Atlas (TCGA): an Immeasurable Source of Knowledge. wo 1A (1A), 68–77. 10.5114/wo.2014.47136 PMC4322527 25691825

[B37] Villani C. (2009). Optimal Transport: Old and New, 338. Berlin: Springer, 23.

[B38] Wang X. Park J. Susztak K. Zhang N. R. Li M. (2019). Bulk Tissue Cell Type Deconvolution with Multi-Subject Single-Cell Expression Reference. Nat. Commun. 10 (1). 10.1038/s41467-018-08023-x PMC6342984 30670690

[B39] Weng L. (2019). From gan to Wgan. arXiv preprint arXiv:1904.08994.

[B40] Yip A. M. Horvath S. (2007). Gene Network Interconnectedness and the Generalized Topological Overlap Measure. Bmc Bioinformatics 8 (1), 22. 10.1186/1471-2105-8-22 17250769 PMC1797055

[B41] Zhang B. Horvath S. (2005). A General Framework for Weighted Gene Co-expression Network Analysis. Stat. Appl. Genet. Mol. Biol. 4 (1), Article17. 10.2202/1544-6115.1128 16646834

[B42] Zhang S. Y. (2021). “A Unified Framework for Non-negative Matrix and Tensor Factorisations with a Smoothed Wasserstein Loss,” in Proceedings of the IEEE/CVF International Conference on Computer Vision, 4195–4203.

