# Supplementary material for: DecOT: Bulk Deconvolution With Optimal Transport Loss Using a Single-Cell Reference
Source: Front Genet. 2022 Feb 4;13:825896. doi: 10.3389/fgene.2022.825896 (PMC8855157; doi:10.3389/fgene.2022.825896)
Supplement: Supplementary file 1 [file DataSheet1.docx]

Supplementary Material

# Supplementary Tables

Supplementary Table 1. The number of cells of each cell type in 54_male dataset.

| cell type | delta | ductal | acinar | beta | alpha |
| --- | --- | --- | --- | --- | --- |
| number of cells | 1 | 5 | 16 | 18 | 85 |

# Supplementary Figures


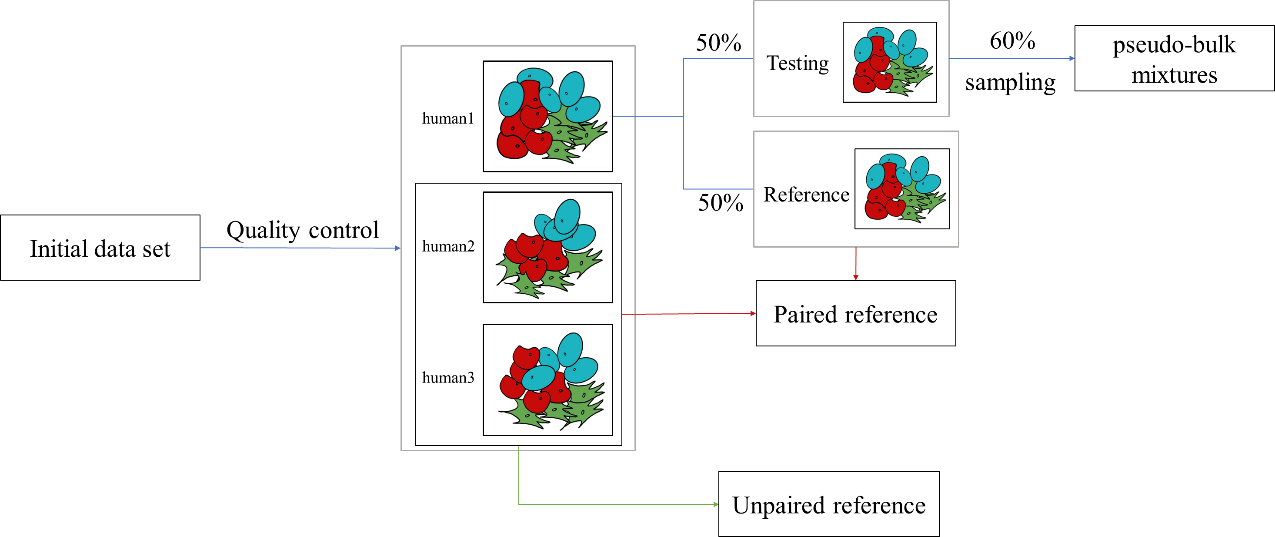


Supplementary Figure 1. Flow chart of constructing pseudo-bulk mixtures. First, we perform quality control on the initial data set. After that, for each individual in the data set, we split their cells into balanced reference and testing datasets with a similar distribution of cell types. Then we generate 200 pseudo-bulk mixtures by randomly sample 60% cells each time in testing datasets and aggregate the expression counts of each gene. The “paired” means that the same individuals as those in bulk mixtures are included in the single cell references. The “unpaired” means that the reference does not contain cells from the same individual of the bulk mixture.


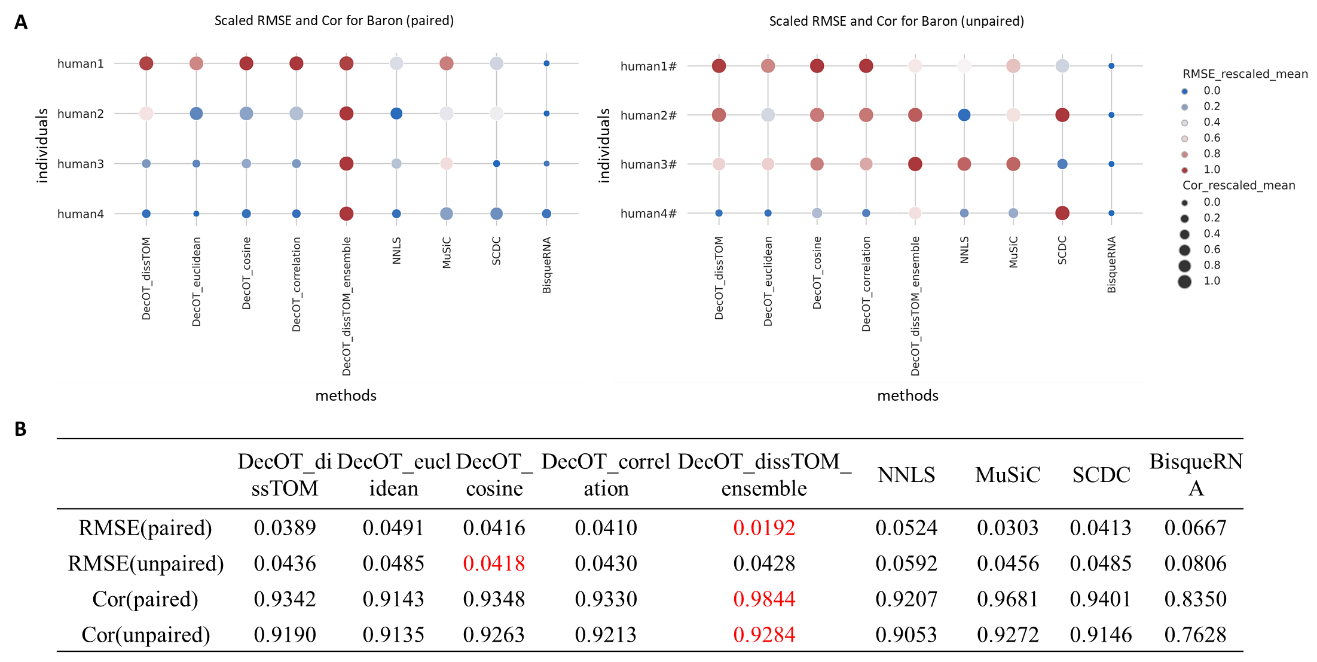


**Supplementary Figure 2.** Benchmark results using dataset Baron. (A) Overview of deconvolution results of individual pseudo-bulk mixtures in Baron dataset. For each individual, we rank the results across different methods and rescale them to the interval between 0 and 1. A darker red and larger point within a line represents a smaller RMSE and a larger Corr. Both of paired(left) and unpaired(right) situations are considered. (B) The average RMSE and Cor of the deconvolution results of all mixtures in dataset Baron. From the overall results, the estimation of DecOT with ensemble framework has smaller errors and stronger correlation than other methods.


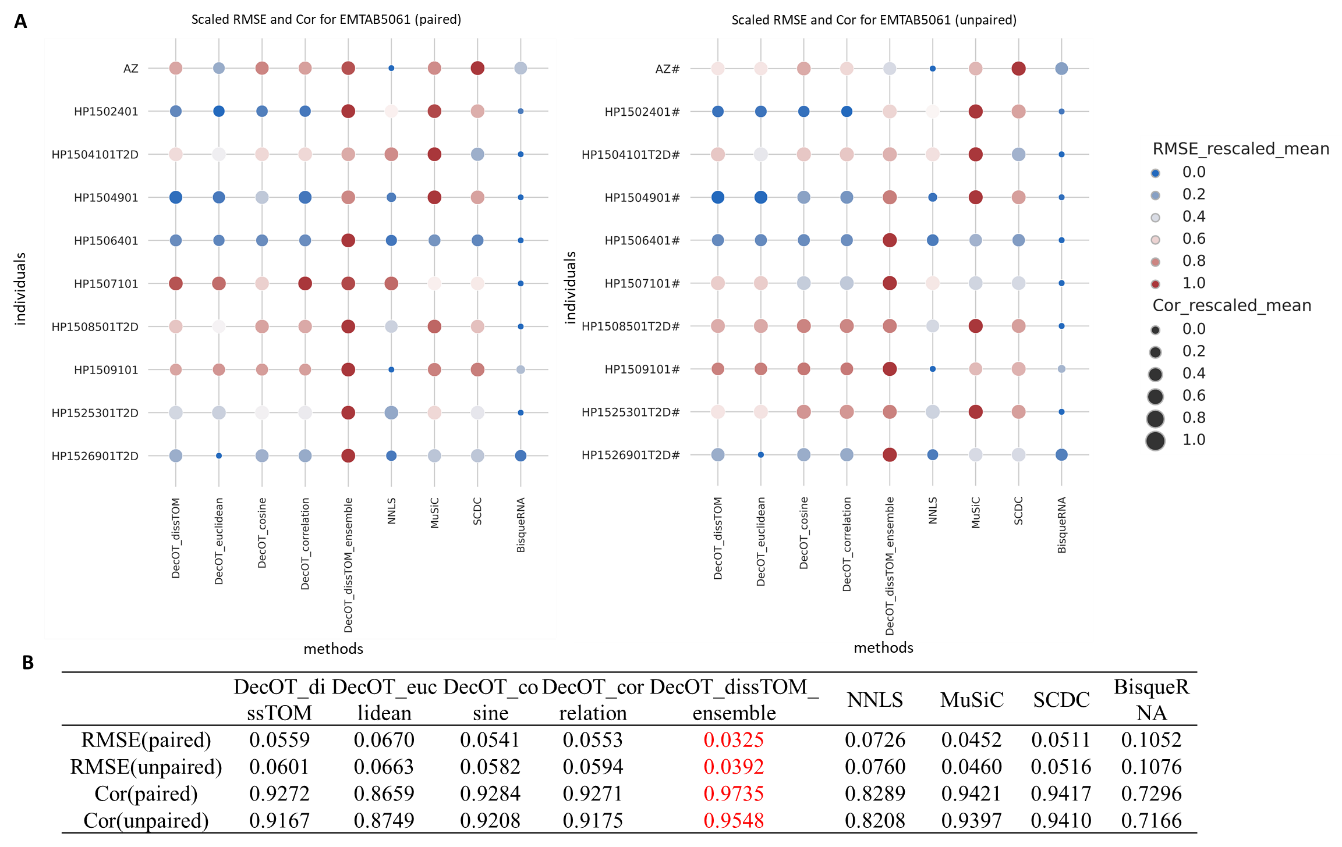


Supplementary Figure 3. Benchmark results using dataset EMTAB5061. (A) Overview of deconvolution results of individual pseudo-bulk mixtures in EMTAB5061 dataset. (B) The average RMSE and Cor of the deconvolution results of all mixtures in dataset EMTAB5061. From the overall results, the estimation of DecOT with ensemble framework has smaller errors and stronger correlation than other methods.


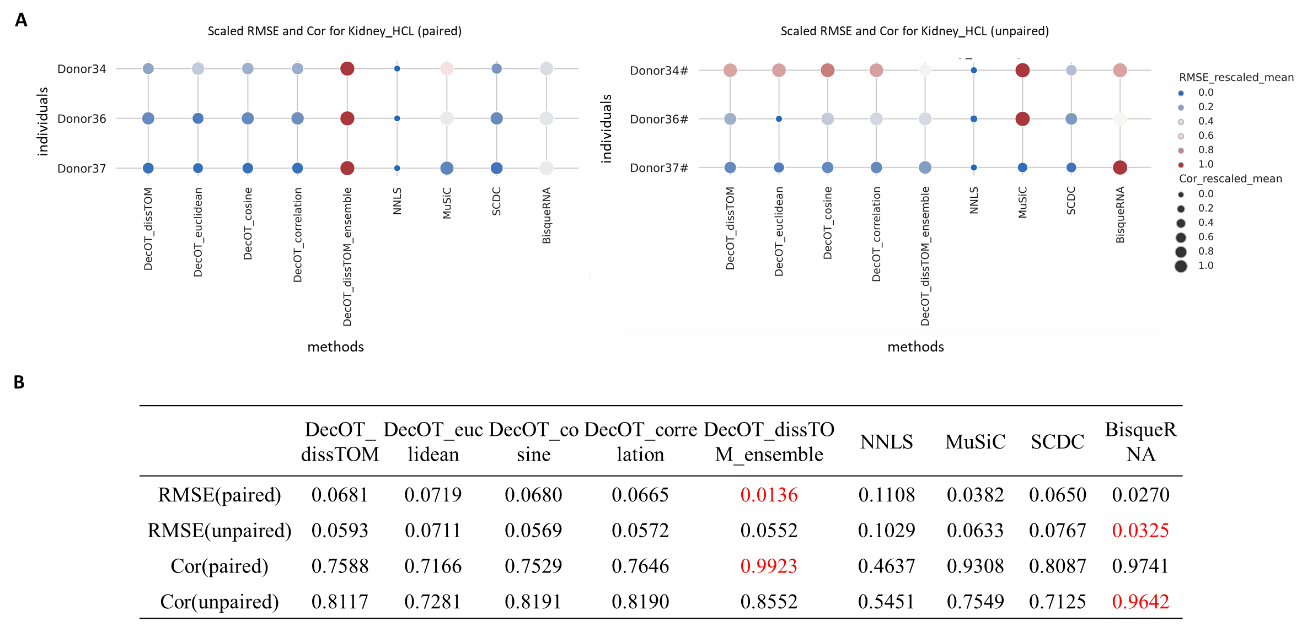


Supplementary Figure 4. Benchmark results using dataset Kidney_HCL. (A) Overview of deconvolution results of individual pseudo-bulk mixtures in Kidney_HCL dataset. (B) The average RMSE and Cor of the deconvolution results of all mixtures in dataset Kidney_HCL. From the overall results, the estimation of DecOT with ensemble framework has smaller errors and stronger correlation than other methods in the case of paired. In the case of unpaired, the effect of DecOT is second only to BisqueRNA.


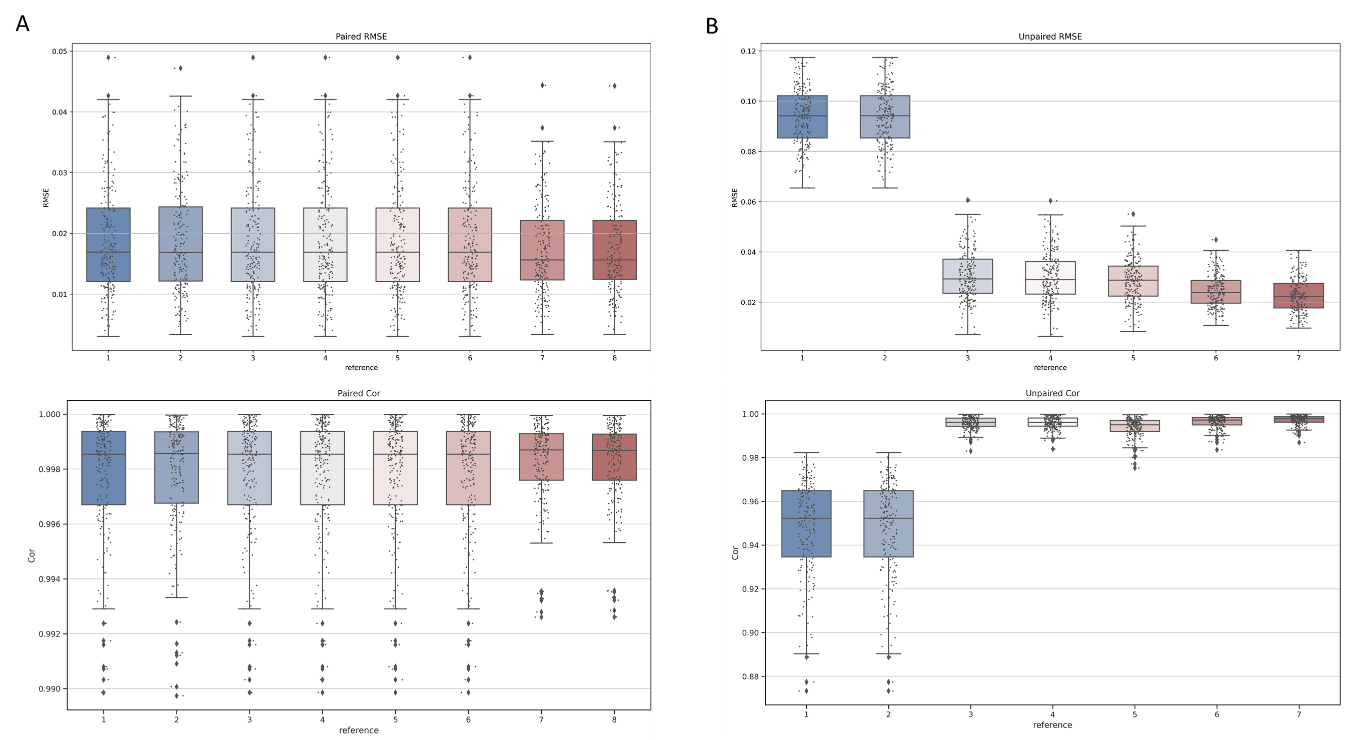


Supplementary Figure 5. Deconvolution results of DecOT as the individual references increases. We analyze artificial pseudo-bulk mixtures of 54_male in GSE81547. (A) For “paired” situation, we start with 54_male cells as reference and gradually add references from other individuals (according to alphabetic order of sample names). (B) For “unpaired” situation, we gradually add reference from other individuals. Including more individual references under the ensemble framework tends to improve the performance of deconvolution in both “paired” and “unpaired” situation.


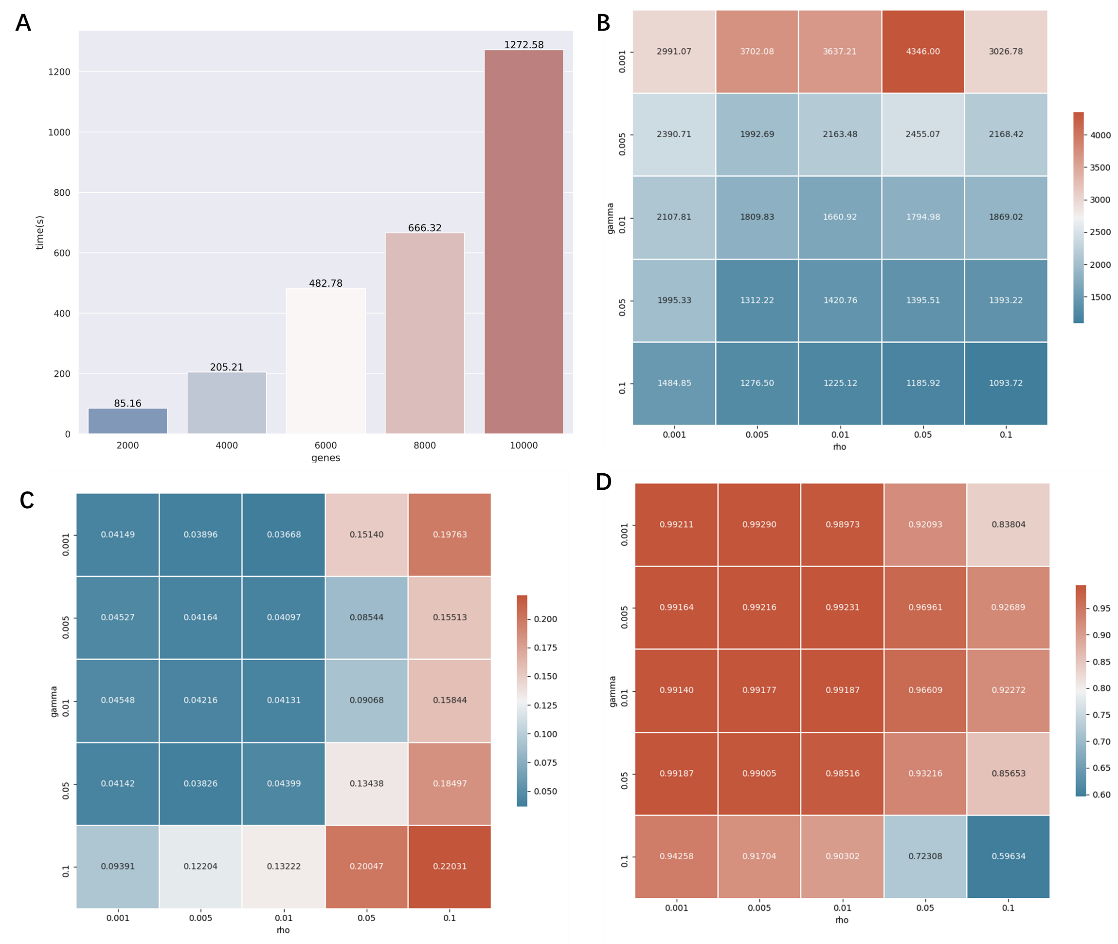


Supplementary Figure 6. Analysis of DecOT's running time and accuracy under different numbers of genes and parameters. (A) The running time of DecOT under different numbers of genes. (B) The running time of DecOT under different choices of two regularization parameters gamma and rho. (C) The RMSE of the estimated result of DecOT under different choices of the two regularization parameters $\boldsymbol{\gamma}$ and $\boldsymbol{\rho}$. (D)The correlation coefficient of the estimated result of DecOT under different choices of the two regularization parameters $\boldsymbol{\gamma}$ and $\boldsymbol{\rho}$. The reduction of the two regularization parameters will make the estimation of DecOT more accurate, and will also increase the required calculation time. In general, DecOT obtains relative accurate results with $\boldsymbol{\gamma}\boldsymbol{\leq}\boldsymbol{0.05}$ and $\boldsymbol{\rho}\boldsymbol{\leq}\boldsymbol{0.01}$.
